# A Method to Address Differential Bias in Genotyping in Large-Scale Association Studies

**DOI:** 10.1371/journal.pgen.0030074

**Published:** 2007-05-18

**Authors:** Vincent Plagnol, Jason. D Cooper, John A Todd, David G Clayton

**Affiliations:** Juvenile Diabetes Research Foundation/Wellcome Trust Diabetes and Inflammation Laboratory, Department of Medical Genetics, Cambridge Institute for Medical Research, University of Cambridge, Cambridge, United Kingdom; University of Alabama at Birmingham, United States of America

## Abstract

In a previous paper we have shown that, when DNA samples for cases and controls are prepared in different laboratories prior to high-throughput genotyping, scoring inaccuracies can lead to differential misclassification and, consequently, to increased false-positive rates. Different DNA sourcing is often unavoidable in large-scale disease association studies of multiple case and control sets. Here, we describe methodological improvements to minimise such biases. These fall into two categories: improvements to the basic clustering methods for identifying genotypes from fluorescence intensities, and use of “fuzzy” calls in association tests in order to make appropriate allowance for call uncertainty. We find that the main improvement is a modification of the calling algorithm that links the clustering of cases and controls while allowing for different DNA sourcing. We also find that, in the presence of different DNA sourcing, biases associated with missing data can increase the false-positive rate. Therefore, we propose the use of “fuzzy” calls to deal with uncertain genotypes that would otherwise be labeled as missing.

## Author Summary

Genome-wide disease association studies are becoming more common and involve genotyping cases and controls at a large number of SNP markers spread throughout the genome. We have shown previously that such studies can have an inflated false-positive rate, the result of genotype calling inaccuracies when DNA samples for cases and controls were prepared in different laboratories, prior to genotyping. Different DNA sourcing is often unavoidable in the large-scale association studies of multiple case and control sets. Here we describe methodological improvements to minimise such biases. These fall into two categories: improvements to the basic clustering methods for calling genotypes from fluorescence intensities, and use of “fuzzy” calls in association tests in order to make appropriate allowance for call uncertainty.

## Introduction

Genome-wide association (GWA) studies are becoming more common because of rapid technological changes, decreasing costs and extensive single nucleotide polymorphism (SNP) maps of the genome [1,2]. However, a major technological challenge is the fact that this ever-increasing number of SNPs is necessarily reliant on fully automated clustering methods to call genotypes. Such methods will inevitably be subject to errors in assigning genotypes because the clouds of fluorescence signals are not perfectly clustered and vary according to many factors, including experimental variation and DNA quality [3]. As it is no longer practical to inspect each genotype call manually, identification of unreliable calls requires a measure of clustering quality. Failure to identify such SNPs leads to an increased false-positive rate and, if a crude quality score is applied, loss of data. Adapting the clustering algorithm to allow for clustering variation arising from the study design can reduce the number of unreliably called SNPs and can minimise the false-positive rate.

The decreasing genotyping costs of GWA studies is permitting the use of larger sample sizes. An efficient design to limit the blood sample collection and genotyping costs is the use of a common control group for several case collections [2]. To this end, the 1958 British Birth Cohort (1958 BBC), an ongoing follow-up study of persons born in Great Britain during one week in 1958 (National Child Development Study), has been used to establish a genetic resource [4] (www.b58cgene.sgul.ac.uk). The Wellcome Trust Case-Control Consortium (WTCCC) has adopted such a design utilising the 1958 BBC and additional blood donors (www.wtccc.org.uk) as a common control group for case collections of seven different diseases. A drawback of this approach is that it can generate a differential bias in genotype calling between case and control DNA samples that originated from different laboratories [3]. This leads to an increased false-positive rate.

In this paper, we compare the genotype calls of a type 1 diabetes (T1D) GWA study using the original clustering algorithm [5] implemented for this genotyping platform and a new algorithm adapted to take into account differential bias in genotype scoring. This study consists of 13,378 nonsynonymous SNPs (nsSNPs) in 3,750 T1D cases and 3,480 1958 BBC controls using the highly multiplexed molecular inversion probe (MIP) technology [6,7]. Previously, we found that the original clustering algorithm [5] performed well when the genotypes clouds were perfectly clustered. However, when variability in the fluorescent signal caused the clouds to be less distinct, we found that a differential bias between cases and controls increased the false-positive rate [3]. The cause of this problem was attributed to the different sources for controls and cases DNA samples that resulted in different locations for the genotyping clouds of fluorescent signal. We addressed this issue by scoring separately cases and controls [3]. We also explored surrogate measures of clustering quality and employed stringent cut-offs to reduce the false-positive rate and extended the concept of genomic control by applying a variable downweighting to each SNP. However, neither approach was optimal, particularly the use of stringent cut-offs, which resulted in a considerable loss of data.

Here, we adapted the methodology to address differential bias between cases and controls in a GWA study. There are three main improvements. Two modifications concern the genotyping algorithm: we used a new scoring procedure that enables cases and controls to be scored together and we adopted a more robust statistical model. The third modification was to use “fuzzy” calls in association tests in order to deal appropriately with call uncertainty. This avoids bias introduced by treating uncertain calls as “missing” when the proportion of such missing calls vary between cases and controls. We also propose a quality-control score for the clustering. These improvements allowed us to significantly increase the number of SNPs available for analysis and to improve the overall data quality. These modifications are generic and can be incorporated into any clustering-based genotyping algorithm. We illustrate this point by applying our algorithm to score the WTCCC control samples (www.wtccc.org.uk), which were generated using the Affymetrix 500K (http://www.affymetrix.com).

## Results

### Genotyping Procedure

Our genotyping procedure follows the original algorithm [5] in fitting a mixture model using the expectation maximization (EM) algorithm but we modified this approach to address the characteristics of our dataset. The original algorithm transformed the two-dimensional fluorescent signal intensity plot into a one-dimensional set of contrasts (see Methods). A mixture of three Gaussian (one heterozygous cloud and two homozygous clouds) was fitted to this one-dimensional set of contrasts using the EM algorithm [8] and data points were assigned to clusters. Data points that could not be attributed to a cluster with high posterior probability were treated as missing data. In addition to the parameters that described the location of the genotyping clouds the model also estimated the *a priori* probabilities (Φ_1_, Φ_2_, Φ_3_) for each cluster; these correspond to the genotype relative frequencies.

**Figure 1:**
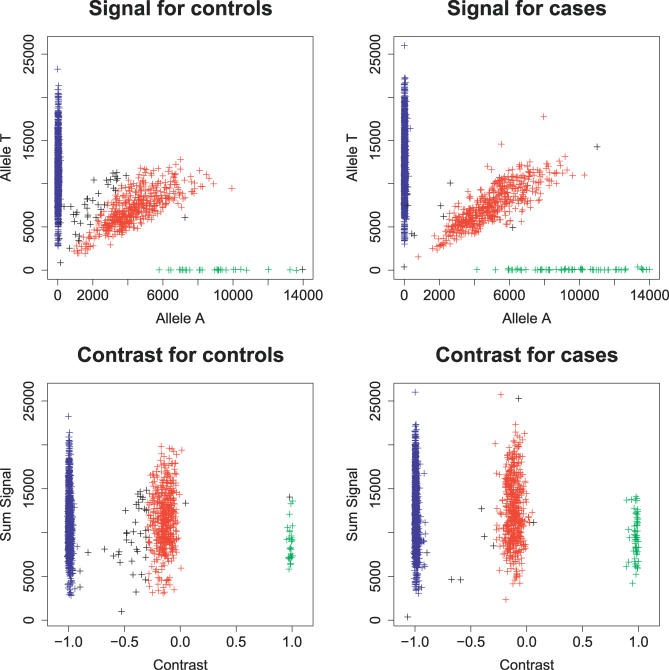
Example of Biased Association Statistic Resulting from Missing Data in the MIP nsSNPs Dataset The top row shows the normalised fluorescent signal intensities for both alleles. The bottom row shows the contrasts (*x*-axis) plotted against the sum signal (*y*-axis). Clustering is based on the original Moorhead et al. [5] algorithm: blue and green crosses belong to both homozygous clouds, red to the heterozygous cloud and black indicates missing calls. The *p*-value for the association test is 0.036 using the original Moorhead et al. [5] algorithm and 0.55 using our modified procedure (which does not label any of the calls as missing).

As control and case DNA samples were processed in different laboratories, the location of the genotyping clouds for the fluorescent signal can differ between cases and controls (see Figure 1). Previously, we scored cases and controls separately to allow for such differences [3]. However, this solution is not ideal. While the location of the clusters can differ, the *a priori* frequencies should be identical in cases and controls under the null hypothesis of no association. Statistical theory shows that the most powerful test is obtained when the maximum likelihood for the nuisance parameters (here the genotyping parameters) is estimated under the null hypothesis. Letting these values differ between cases and controls resulted in overestimated differences in allele frequencies and increased over-dispersion of the test statistic. Our modified algorithm linked the clustering for cases and controls by assuming genotype frequency parameters to be identical but imposed no such restriction on the location of the genotyping clouds. Variability in allele frequencies across geographic regions is also allowed. We extended this approach to score nsSNPs on the X chromosome to account for male/female copy number differences (see Methods).

In the original algorithm, the *a priori* frequencies for the three clusters (Φ_1_, Φ_2_, Φ_3_) are linked by the condition Φ_1_ + Φ_2_ + Φ_3_ = 1, leaving two free parameters. We investigated the effect of further constraining these frequencies to be consistent with Hardy-Weinberg equilibrium (HWE). In that version of the algorithm, the *a priori* frequencies (Φ_1_, Φ_2_, Φ_3_) are parameterised as (π^2^,2π(1 − π),(1 − π)^2^) using a unique parameter π.

We also found that the statistical model for the fluorescent clouds was not robust to excessive variability of the fluorescent signal within a genotyping cloud. Because our association tests require that no data point is treated as missing (see below), we needed a model robust to outliers. As the tails of the Gaussian distribution decay too fast, we replaced the Gaussian distributions with *t*-distributions. Our parameter inference procedure (EM algorithm, see [8]) uses a representation of the *t*-distributions as a Gaussian random variable with a variance sampled randomly from a Gamma distribution. Fortunately, the sample size of this study was sufficient to estimate these additional parameters.

### Association Test

The nsSNPs were analysed using the one degree of freedom Cochrane-Armitage trend test [9]. In this statistical framework, the outcome variable is the disease phenotype and the explanatory variable is the genotype. The null hypothesis is the absence of effect of the genotype on the odds of developing the disease. This test statistic for association is a score test; the score statistic is the first derivative of the log-likelihood of the data at the null value of the parameter tested. The test statistic is obtained by dividing the score test by its variance under the null, derived using a permutation argument (see Methods). We also used a stratified version of this test introduced originally by Mantel [10] that allows for variability in allele frequency and disease prevalence across 12 broad regions in Great Britain [3]. In this version of the test, the score and its variance are summed over the 12 strata to obtain the overall score and variance. The ratio of the square of the score statistic to its variance is asymptotically distributed as a χ^2^ random variable with one degree of freedom

We explored how differential bias could affect the distribution of the test statistic. An aspect of the data that is affected by the differential bias is the frequency of missing calls and the way these missing calls affect the genotyping clouds. These differences increased the over-dispersion level (see for example Figure 1). We found that the best solution was to avoid the use of missing calls and call all available samples, making appropriate allowance for call uncertainty. This led us to modify the association test. To do so, we reformulated the association test as a missing data problem in which the distribution of the genotypes status is estimated conditionally on the fluorescent signal and the geographic origin of the sample (see Methods). This modification of the test amounts to replacing the score statistic with its expectation under this posterior distribution of the genotype status. Similar ideas have been used in the context of haplotype phasing [11].

### Simulation Study

The elevated rate of false positives observed in the data resulted from an over-dispersion of the test statistic. We estimated the over-dispersion factor, λ, by calculating the ratio of the mean of the smallest 90% of the observed test statistics to the mean of the smallest 90% of the values expected under the null hypothesis of no association [3]. Using the smallest 90% is motivated, in a case-control framework, by the exclusion of the “true” associations that are caused by actual differences between cases and controls and that can significantly affect the mean value of the test statistic. To make the interpretation of the results easier, we report Δλ, the difference between the theoretical over-dispersion factor (equal to 1) and the observed one: a value of 1% for Δλ means that the over-dispersion factor λ is 1.01.

We illustrate the impact of our modifications by analysing simulated fluorescent signal data. We used two models for the quality of the fluorescent signal (high and low quality SNPs). We considered various scenarios for the minor allele frequency in cases and controls and simulated 100,000 SNPs for each scenario. The signals were scored in three different ways: (1) the full algorithm, as described above; (2) cases and controls were called separately; and (3) fuzzy calls were not used. In (3), we assigned a probability 1 to the most probable call under the posterior distribution and we called a sample missing when the probability of this most probable call was less than 0.95. For each version of the scoring algorithm, we report Δλ under the null hypothesis of no association (i.e., identical population frequencies in cases and controls). We also compared the power for the three versions of the algorithm. Following Neyman-Pearson's lemma [12], the best test is the one that, for a given type 1 error (the probability to reject the null when the null is true), has the lowest type 2 error (the probability to accept the null when the alternate hypothesis is correct). In practice it implied correcting the test statistic for the over-dispersion and estimating the fraction of the SNPs simulated under the alternate hypothesis for which the null hypothesis was accepted. We set the type 1 error to 0.05 in our simulations. Results are reported in Table 1.

**Table 1:**
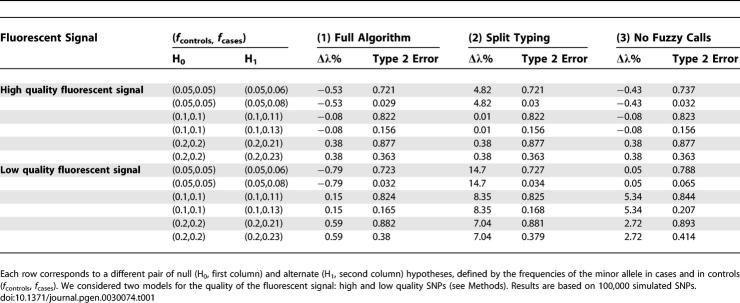
Over-Dispersion Factor Δλ (Estimated under the Null ) and Type 2 Error for Three Versions of Our Algorithm: Full Method (1), without the Joint Typing (2), and without the Use of Fuzzy Calls (3)

We found that, as expected, all three versions of the algorithm performed comparably well when the quality of the fluorescent signal was high. In that situation, the only situation where the level of over-dispersion was significant was the separate typing procedure combined with low minor allele frequency. Clustering based algorithms are not well suited to estimate parameters when the number of data points in a genotyping cloud is low and this weakness was amplified when cases and controls were called separately.

However, strong differences appeared for the lower quality SNPs. We found that there was little over-dispersion when the full algorithm was used (Δλ between −0.79% and 0.59%). However, when the split typing version was used, over-dispersion ranged from 7.04% to 14.7%, increasing as the minor allele frequency decreases. In addition, comparison between joint and split typing methods showed that the power of the study (measured by the type 2 error) was very similar. However, this observation is misleading, as when the data consists of a mixture of high and low quality SNPs, applying a constant correction factor independently of the fluorescent signal quality would result in a loss of power for the split typing method. For the full method, we found a near perfect agreement between theoretical and observed distributions, and the use of a correction factor was not necessary.

Not using the fuzzy calls had a less obvious effect on the over-dispersion. As mentioned above, the high quality SNPs were not affected because the vast majority of calls was certain. For the low quality fluorescent signal model, we found that on average 1.2% of the individuals had a probability of the most likely call lower than 0.95. Labelling these unclear calls as missing significantly affected the over-dispersion slope, which reached a maximum at intermediate frequencies (Δλ = 5.34% at minor allele frequency 10%, see Table 1). In addition, and unlike the split typing version of the algorithm, the type 2 error increased significantly (between 1% and 5%). We also note that calls with a most likely probability lower than 70% were rare (on average 0.4% of the calls). Therefore, replacing the fuzzy posterior distribution with the most likely call had almost no effect on the over-dispersion slope, indicating that for the range of model and data considered here the inclusion of fuzzy calls is not critical as long as missing calls are not used.

### MIP nsSNPs Dataset

The MIP data consisted of 13,378 nsSNPs typed in 3,750 cases and 3,480 controls. We analysed 11,579 nsSNPs with minor allele frequency estimated to be greater than 0.01. We also excluded 281 nsSNPs in the HLA region that is known to be associated with T1D, leaving 11,298 nsSNPs.

Initially, using the original calls, we employed stringent cut-offs for the surrogate measures of clustering quality: case and control call rates both greater than 95%, difference in call rates between controls and cases smaller than 3% and HWE χ^2^ < 16. This resulted in 2,079 high-quality nsSNPs with an over-dispersion factor Δλ of 4.5%. We obtained a lower over-dispersion of 1.5% when these nsSNPs genotypes were called using the adapted algorithm. As expected, this difference in over-dispersion between algorithms became more marked as less stringent cut-offs were applied. For example, lowering the call rate cut-off to 90% resulted in 5,294 nsSNPs with an over-dispersion of Δλ = 17% using the original Moorhead et al. [5] scoring algorithm and 8.1% using the adapted algorithm on the same set of nsSNPs.

**Figure 2:**
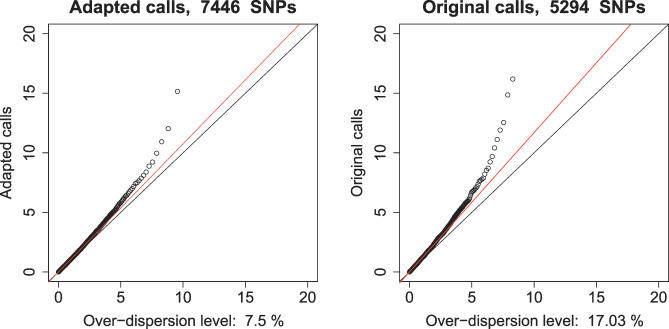
Quantile–Quantile Plot Comparing the Observed Distribution of the Association Statistic (*y*-Axis) with the Predicted Distribution under the Null (*x*-Axis) The leftmost graph uses our set of calls for our best 7,446 nsSNPs and the rightmost graph relies on the original calls for the best 5,294 nsSNPs in 3,750 cases and 3,480 controls.

We propose a measure of clustering quality that compares the variability of the signal within a cluster with the variability between clusters (see Methods). The lower limit for the quality measure was set such that beyond this value the over-dispersion factor λ remained constant. When we selected the nsSNPs according to our quality-control measure this resulted in 7,446 nsSNPs with an over-dispersion slope of 7.5% using our improved algorithm (Figure 2). For the same set of SNPs the over-dispersion level was 21% using the original calls.

We investigated the effect of our modifications by scoring the data using various configurations of the algorithm and the association test. The quality was measured using the level of over-dispersion Δλ of the test statistic for the stratified test (see Table 2). For the genotyping procedure, we found that the split clustering of cases and controls significantly increased the over-dispersion level: Δλ = 10.5%, +3% compared to the joint typing of cases and controls with a unique set of *a priori* frequencies (Δλ = 7.5%). However, letting these *a priori* frequencies vary across geographic regions in the stratified version of the test did not change the results, although a stronger discrepancy might have been observed if cases had not been well matched geographically with the controls. Assuming a Gaussian model (rather than *t*-distribution in the adapted algorithm) also significantly increased the over-dispersion level (+4.3%).

**Table 2:**
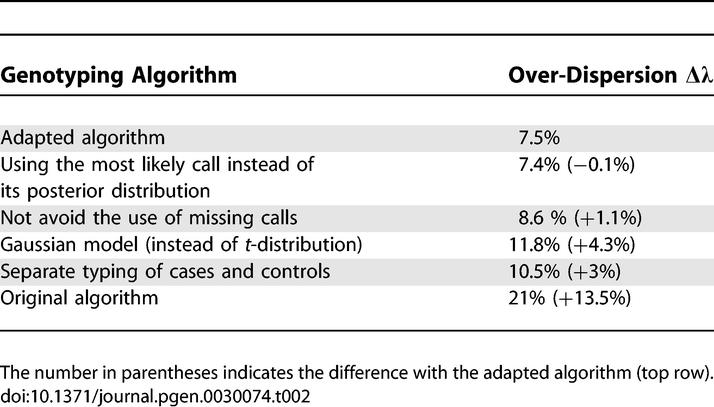
Impact of Our Modifications on the Over-Dispersion Measure for the 7,446 nsSNPs That Passed our Quality Threshold in 3,750 Cases and 3,480 Controls

Imposing the *a priori* frequencies to be consistent with HWE did not lower the over-dispersion level (+1.5%), probably because this condition was too stringent. We investigated a weaker version of this constraint in the parameter estimation: we first estimated the parameters under the HWE constraint. Then we relaxed this assumption in the second step but used the parameter values estimated in the first step as a starting point for the iterative parameter estimation procedure. This modification also did not lower the over-dispersion level (Δλ = 8.2%, +0.7% compared to the adapted calls). However, while this further constrain did not improve the over-dispersion overall, this two-step procedure helped with finding the global maximum of the likelihood function for a small fraction of nsSNPs for which the variance of the fluorescent signal was large. Therefore, it provided an alternative scoring method useful to maximise the number of typed nsSNPs while increasing the over-dispersion only slightly.

Regarding the association test, we investigated the effect of missing calls. For each nsSNP, we called a sample missing when the probability of belonging to the most likely genotype cloud was less than 95%. The number of missing calls varied greatly across nsSNPs: the median of the average number of missing calls across the nsSNPs that pass the quality threshold is 0.2% but this median number is 1% among the 2,171 nsSNPs with the lowest quality score among the best set of 7,446 nsSNPs. We found that the use of missing calls slightly increased the level of over-dispersion (+1.1% compared to the same algorithm in the absence of missing calls). However, missing calls have a larger effect on the quality scores: re-estimating a best set of 7,446 nsSNPs but computing the quality scores with missing data generated an over-dispersion of 10.5%. This larger over-dispersion is explained by the fact that introducing missing calls biased the computation of the quality scores and prevented us from identifying low quality nsSNPs (see Discussion). However, once we avoided the use of missing calls and called all available samples, using the most likely call instead of the posterior distribution had little effect (Δλ = 7.4%, 0.1% lower than our adapted calls). This limited effect is expected because split calls are rare for the range of models we considered.

### WTCCC Control Dataset

In this section, we show the result of our adapted algorithm applied to a different genotyping platform, the Affymetrix Mapping 500K array set. These data have been generated by the WTCCC (www.wtccc.org.uk). The WTCCC is a GWA study involving seven different disease groups. For each disease, the WTCCC genotyped 2,000 individuals from England, Scotland, and Wales. Disease samples will then be compared to a common set of 3,000 nationally ascertained controls also from the same regions. These controls come from two sources: 1,500 are representative samples from the 1958 BBC and 1,500 are blood donors recruited by the three national UK Blood Service. Here, we compare the WTCCC control groups. This comparison is interesting because in a typical GWA study, we expect a fraction of the over-dispersion to reflect actual genetic differences between control and disease groups. However, when comparing two sets of healthy controls the interpretation of the results is easier, as both groups should be representative samples of the population.

**Figure 3:**
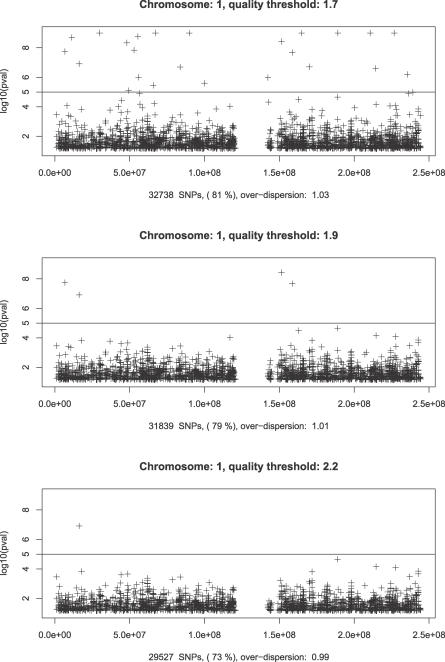
Distribution of *p*-Values for the Association Test between the 1958 BBC Samples and the UK Blood Donors (WTCCC Control Dataset) for Three Different Quality Thresholds

We first show that our quality measure was efficient at distinguishing poorly typed SNPs from correctly typed ones. We illustrate this point by showing the distribution of *p*-values on Chromosome 1 for three quality thresholds (see Figure 3). Because the distribution of the fluorescent signal differs between the MIP platform and the Affymetrix 500K, the optimum threshold also differs. We found that approximately 79% of the SNPs have a minor allele greater than 0.01 as well as a quality score greater that 1.9. Given a total number of 40,220 SNPs on this chromosome, is approximately the level beyond which no *p*-value is expected. For that quality threshold of 1.9, only four SNPs are obvious false positives with *p*-values beyond 1 × 10^−5^. Visual inspection of the clusters confirmed that these were indeed clustering errors. When we increased the quality score to 2.2, only one of the four SNPs remained (with a quality score of 2.3). As approximately 12% of the Affymetrix 500K SNPs are monomorphic in the British population, we found that only 9% of the SNPs did not pass our quality threshold, while keeping the false-positive rate close to zero. Similar numbers were found on other chromosomes.

In addition, we compared our algorithm with the BRLMM calls, commonly used on this platform and provided by Affymetrix. For each autosomal chromosome we used BRLMM and our adapted algorithm to select the subset of SNPs with a quality score greater than 1.9 and a minor allele frequency greater than 0.01. For both sets of calls, we computed the fraction of SNPs that pass that threshold. In order to make results comparable, we calculated the over-dispersion slope for the SNPs that passed both the BRLMM and the adapted calls threshold (see Table 3). We found that the percentage of SNPs that pass the quality threshold is typically 4% higher using our adapted algorithm, while the over-dispersion remained 2%–5% lower, indicating a significant improvement.

**Table 3:**
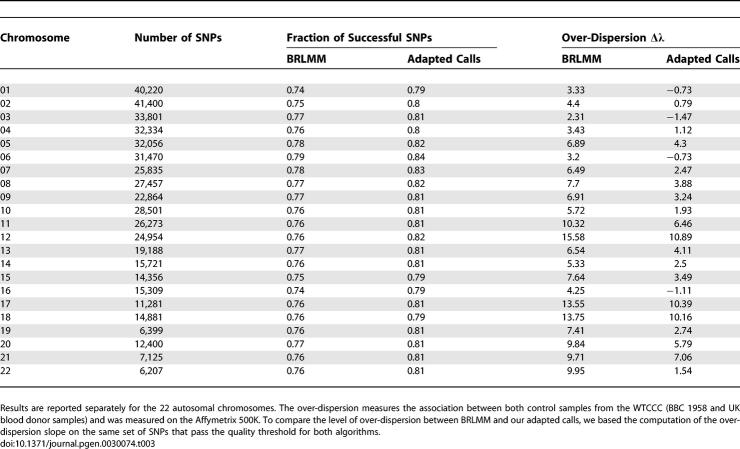
Level of Over-Dispersion for the SNPs That Pass Both the Minor Allele Frequency Cut-Off (Greater than 0.01) and the Quality Threshold of 1.9

## Discussion

In this T1D nsSNP GWA study, the adapted algorithm was successful at scoring more nsSNPs confidently (7,446 nsSNPs instead of 5,294 nsSNPs) and, as a consequence, reducing the false-positive rate: over-dispersion decreased from 17% to 7.5%. Rather than developing an entirely new genotyping algorithm we have adapted the current algorithm for GWA with the motivation of controlling the false-positive rate resulting from a cases/controls genotyping bias. Consequently, these modifications are relevant to all clustering based genotyping algorithms. Here, we considered the MIP genotyping technology [7] and the Affymetrix 500K array, but these modifications are also applicable to the Illumina platform (http://www.illumina.com). Our results show that the most important recommendation consists of scoring the different datasets (typically cases and controls) in a centralised manner, when this is possible. Introducing fuzzy calls is less important as long as one avoids the use of missing calls.

In practice, a key component of any genotyping algorithm is the ability to provide a single measure of clustering quality. Previously, we used surrogate measures of clustering quality (such as call rate and deviation from HWE) to identify unreliable SNPs, but this approach was not optimal [3]. Our measure of clustering quality compared the locations of the clusters of fluorescent signals with the variability of this signal within a cluster. However, to be really informative, this measure should be computed in the absence of missing calls. Excluding calls artificially reduces the variability of the signal within each cloud and biases the quality measure upward. Contrary to intuition, when using the calls provided by the original MIP algorithm [7] to compute both the quality measure and the association statistic, the over-dispersion level is higher for the nsSNPs that have the highest confidence value: Δλ = 26% for a confidence greater than 8 (1,116 nsSNPs) and Δλ = 15% for a confidence level between 5 and 8 (2,393 nsSNPs). Visual inspection of the clustering for these nsSNPs showed that such high confidence levels were typically associated with small variability of the fluorescent signals within clouds. In that situation, the original algorithm called missing those data points located a few standard deviations away from the center of the cluster. When these missing calls occurred differently in cases and controls it resulted in an increased over-dispersion of the association statistic (such as in Figure 1).

We note that in spite of our efforts a level of over-dispersion remains even for the 2,079 nsSNPs with near perfect clustering (Δλ = 1.5%). This estimate is noisy and its significance or causes are difficult to assess. However, we note that in the larger set of 7,446 nsSNPs, the inclusion of 21 non-Caucasian samples increased the over-dispersion from 7.5% to 12.1%. Also, if there were any undetected close relations in the collections of cases and controls this could also increase the level of over-dispersion (we did ensure that inadvertent or deliberate sample duplications were removed, and no first-degree relatives were included in the study)

The difference between the lower bound of 1.5% (in the high quality set of 2,079 nsSNPS) and our 7.5% level (in the larger set of 7,446 nsSNPs) is probably associated with remaining imperfections in our statistical model. As pointed out in the Results section, replacing the most likely call with its posterior distribution given the fluorescent signal had little effect on the level of over-dispersion. Indeed, when a data point was located between two clusters, the algorithm did not assign an intuitive 50%/50% probability on both adjacent clouds but rather put a weight close to one on the cloud with the largest standard deviation. This replacement of “grey” calls with “black or white” amplified the difference between cases and controls and contributed to the remaining level of over-dispersion.

## Materials and Methods

### Description of the genotyping algorithm.

The original algorithm is described in [5]. Genotypes are scored based on the contrast measure: for a SNP with alleles A and G and signal intensities *I_A_* and *I_B_*, respectively, *S = I_A_ + I_B_* and *contrast =* sinh(2*I_A_I_G_*/*S*)/sinh(2). In this approach a mixture of three Gaussian is then fitted to the set of contrast values. Three parameters (Φ_1_, Φ_2_, Φ_3_) with the constraint Φ_1_ + Φ_2_ + Φ_3_ = 1 represent the *a priori* probabilities to belong to each of the three clouds (before knowing the value of the contrast). Parameters (*a priori* frequency estimates location μ, and standard deviation σ of the three clouds) are estimated using the EM algorithm [8]. This Gaussian mixture is replaced with *t*-distributions in our modified method. A possible representation of a *t*-distribution with *n* degree of freedom, variance parameter σ and mean μ is the following:

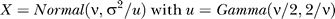



This representation is used in the version of the EM algorithm we used to score the data [8]. It used a data augmentation procedure and treated the variables *u* as missing data.

### Linked clustering of cases and controls.

When controls and cases are typed separately each sample has its own set of parameters Θ: (Φ_1_, Φ_2_, Φ_3_) that describe the *a priori* allele frequencies as well as 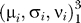 that describe the location of the three genotype clouds. In the linked version of the scoring the *a priori* frequencies (Φ_1_, Φ_2_, Φ_3_) are identical for both samples (cases and controls). In the EM algorithm the set of parameters Θ is estimated iteratively. The estimator of Φ*_i_* at step (*k*+1) is 
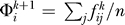
 where *n* is the number of observations and 
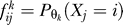
 . When the scoring is done separately for cases and controls this estimator is computed separately for both samples. In the linked version of the scoring this sum is computed jointly for cases and controls. For the stratified association test, each geographic region *s* has its own set of parameters (Φ_1_, Φ_2_, Φ_3_) that is estimated separately for each region, but jointly for cases and controls. The rest of the EM algorithm follows [8].

### Typing of the X chromosome.

We extended our linked clustering approach to deal with nsSNPs on the X chromosome. Because of male/female copy number differences this situation is similar to differential genotyping bias as the location of the genotyping clouds can differ across samples. We extended our linked clustering approach to this situation: the location of the genotyping clouds could differ but the *a priori* frequencies were estimated jointly. In that case we denote 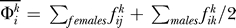 and 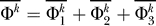 . Then for the female sample we have 
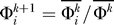
 . For the male sample:

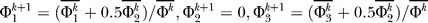



### Imposing HWE.

The linked clustering approach can be extended to impose HWE for the *a priori* frequency estimates (Φ_1_, Φ_2_, Φ_3_). The frequencies are parameterised as (π^2^,2π(1 − π),(1 − π)^2^). Using the same notations the EM estimator becomes: 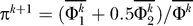 . This approach can also be extended to X chromosome SNPs as presented above.

### Association statistic.

We first consider the unstratified version of the test (see Protocol S1 for a complete derivation of the test). We denote the disease status (the outcome variable in our model) as a vector of binary variables *Y*. The vector *X* of explanatory variables (the genotypes) can take three values (1,2,3). We assume a logistic model: log*it*[*P*(*Y* = 1)] = α + β*X*. The score statistic can be written as:

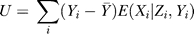



The score variance can be computed using a profile likelihood argument:

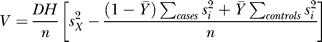
where D and H are the numbers of cases and controls, 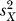 is the sample variance of the expected value of the genotype variable *X,* and 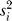 is the variance of *X_i_* under the fuzzy distribution. The test statistic *U* ^2^/*V* is χ^2^ with one degree of freedom under the null.

### Extension for stratification.

The derivation of this test is available in Protocol S1. In that version of the test the score statistic becomes:

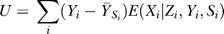
where *S_i_* is the strata for the individual *i* and 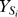 the mean value of *Y* in that strata. Each strata has its own score variance (computed as in the nonstratified situation) and the contribution of each strata is then summed to obtain the overall score variance. The test statistic *U* ^2^/*V* is still distributed as χ^2^ with one degree of freedom under the null.

### Measure of clustering quality.

We designed a measure that captures the intuition that clouds of points are well separated for a given SNP. We use the difference between the centres of adjacent clouds divided by the sum of the standard deviation for these two clouds. Center and standard deviation of the clouds is computed based on the most likely calls. The final quality measure for a SNP is the minimum computed over each pair of clusters. This computation is done for cases and controls separately and the minimum over both samples is then computed. As expected, increasing that threshold is inversely correlated with over-dispersion. The over-dispersion stops decreasing at a threshold of 2.8 and we used this value to generate our set of 7,446 SNPs.

### Simulation details.

When simulating SNPs we simulated directly the set of contrasts. For high quality SNPs, the centres of the three genotyping clouds are −0.9,0,0.9. The three *t*-distributions have degree of freedom equal to ν = 10 and the scaling factor for the standard deviation is 0.03. The standard error for each genotyping cloud is then equal to 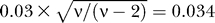 .

For lower-quality SNPs, the centres of the three genotyping clouds are also −0.9,0,0.9. The three *t*-distributions have degree of freedom equal to ν = 3.5 and the scaling factor for the standard deviation is 0.1. The standard error for each genotyping cloud is then equal to 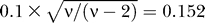 .

## Supporting Information

Protocol S1 Derivation of the Test Statistic
(85 KB PDF)

## Abbreviations

1958 BBC: 1958 British Birth Cohort
EM: expectation maximization
GWA: genome-wide association
HWE: Hardy-Weinberg equilibrium
MIP: molecular inversion probe
nsSNP: nonsynonymous SNP
SNP: single nucleotide polymorphism
T1D: type 1 diabetes
WTCCC: Wellcome Trust Case-Control Consortium
