# Supplementary material for: A Method to Address Differential Bias in Genotyping in Large-Scale Association Studies
Source: PLoS Genet. 2007 May 18;3(5):e74. doi: 10.1371/journal.pgen.0030074 (PMC1868951; doi:10.1371/journal.pgen.0030074)
Supplement: Supplementary file 1 [file pgen.0030074.sd001.pdf]

Protocol S1: Derivation of the test statistic

V. Plagnol, J. D. Cooper, J. A. Todd and D. G. Clayton

Model description

The disease status Y (the outcome variable in our model) is a vector of binary variables. The vector X of explanatory variables (the genotypes) can take three values $(1, 2, 3)$. We assume a logistic model: $\text{logit}\left[\mathbb{P}(Y_i = 1)\right] = \alpha + \beta X_i$. We denote the set of fluorescent intensities by Z.
$\gamma = (\alpha, \beta)$ describe the relation between genotype X and disease status Y. A second set of parameter θ describes the location of the fluorescent signal clouds. A third set ϕ describe the allelic frequencies for the genotype X in this case-control study. The full likelihood can be written as:

$$\mathbb{P}(X, Y, Z|\gamma, \phi, \theta) = \mathbb{P}(X|\phi)\mathbb{P}(Y|X, \gamma)\mathbb{P}(Z|X, Y, \theta)$$

Here, X is a missing data. We note that the dependence of the distribution $\mathbb{P}(Z|X, Y, \theta)$ on Y results from the differential bias (the disease status affects the fluorescent signal).

Non-stratified score test

The score statistic is the derivative of the log-likelihood with respect to β taken at $\beta = 0$. Therefore, the contribution of a single individual to the score is:

$$\begin{aligned}
\frac{\partial \log L(\beta|Z, Y)}{\partial \beta} &= \frac{\partial}{\partial \beta}\left(\log \mathbb{P}(Y, Z|\alpha, \beta, \phi, \theta)\right) \\
&= \frac{\partial}{\partial \beta}\left(\log \sum_{X=1}^{3} \mathbb{P}(Z|X = i, Y)\mathbb{P}(Y|X = i; \alpha, \beta)\mathbb{P}(X = i)\right) \\
&= \frac{\sum \mathbb{P}(X = i)\mathbb{P}(Z|X = i, Y)\frac{\partial}{\partial \beta}\mathbb{P}(Y|X = i; \alpha, \beta)}{\sum \mathbb{P}(Z|X = i, Y)\mathbb{P}(Y|X = i; \alpha, \beta)\mathbb{P}(X = i)} \\
&= \frac{\sum \mathbb{P}(X = i)\mathbb{P}(Z|X = i, Y)\mathbb{P}(Y|X = i; \alpha, \beta)\frac{\partial}{\partial \beta}\left(\log \mathbb{P}(Y|X = i; \alpha, \beta)\right)}{\sum \mathbb{P}(Z|X = i, Y)\mathbb{P}(Y|X = i; \alpha, \beta)\mathbb{P}(X = i)} \\
&= \frac{\sum \mathbb{P}(X = i)\mathbb{P}(Y, Z|X = i; \alpha, \beta)\frac{\partial}{\partial \beta}\left(\log \mathbb{P}(Y|X = i; \alpha, \beta)\right)}{\mathbb{P}(Y, Z|\alpha, \beta)} \\
&= \mathbb{E}_{(X|Y,Z)}\left[\frac{\partial \log \mathbb{P}(Y|X = i; \alpha, \beta)}{\partial \beta}\right]
\end{aligned}$$

We have [1, Chap. 4]:

$$\frac{\partial \log \mathbb{P}(Y|X; \alpha, \beta)}{\partial \beta} = (Y - \pi_X)X$$

where $\pi_X = \mathbb{E}(Y|X;\alpha,\beta)$. Therefore:

$$\mathbb{E}_{(X|Y,Z)}\left[\frac{\partial \log \mathbb{P}(Y|X;\beta)}{\partial\beta}\right] = (Y-\pi_X)\mathbb{E}(X|Y,Z)$$

Replacing α by its MLE at $\beta = 0$ we have $\pi_X = \bar{Y}$ (independent of X) and one obtains the score statistic U by summing over all individuals:

$$U = \sum_i (Y_i - \bar{Y})\mathbb{E}(X_i|Z_i,Y_i)$$

Stratified score test

In the stratified version we define a geographic indicator variable $S_i \in \{1,\ldots,S\}$ and:

$$\text{logit}\left[\mathbb{P}(Y_i = 1)\right] = \alpha + \beta X_i + \sum_s \gamma_s 1_{S_i = s}$$

As in the non-stratified case the contribution of one individual to the likelihood is:

$$\begin{aligned}\frac{\partial \log L(\beta|Y_i,Z_i,S_i)}{\partial\beta} &= \mathbb{E}_{(X_i|Y_i,Z_i,S_i)}\left[\frac{\partial \log \mathbb{P}(Y_i|X_i;\alpha,\beta,\gamma)}{\partial\beta}\right] \\ &= \mathbb{E}_{(X_i|Y_i,Z_i,S_i)}\left[(Y_i - \pi_i)X_i\right] \\ &= (Y_i - \pi_i)\mathbb{E}(X_i|Y_i,Z_i,S_i)\end{aligned}$$

where $\pi_i = \mathbb{E}(Y_i|X_i,S_i;\alpha,\beta)$
Replacing α,γ by MLEs at $\beta = 0$ we have: $\pi_i = \bar{Y}_{S_i}$ where $\bar{Y}_s$ is the average Y in the strata s. When summing over all individual we obtain:

$$U = \sum_i (Y_i - \bar{Y}_s)\mathbb{E}(X_i|Z_i,Y_i,S_i)$$

The presence of the geographic variable Z_i indicates that the scoring algorithm must account for the geographic stratification. In that test each stratum has a score (computed as in the non-stratified case) and the overall score is the sum over strata. The score variance is also computed separately for each stratum (as in the non-stratified case) and then summed over strata. As in the non-stratified case the test statistic U^2/V is distributed as chi-square with one degree of freedom under the null.

Computation of the score variance

Profile likelihood argument
We derive the score variance using a profile likelihood argument. The score variance is the inverse of the marginal value (in β) of the inverse of the information matrix. Considering only the logit model, the information matrix is:

$$I(\alpha,\beta) = \begin{pmatrix} \frac{\delta^2 \log L(Y,X|\alpha,\beta)}{\delta\beta^2} & \frac{\delta^2 \log L(Y,X|\alpha,\beta)}{\delta\beta\delta\alpha} \\ \frac{\delta^2 \log L(Y,X|\alpha,\beta)}{\delta\beta\delta\alpha} & \frac{\delta^2 \log L(Y,X|\alpha,\beta)}{\delta\alpha^2} \end{pmatrix} = \pi_{X_i}(1-\pi_{X_i})\begin{pmatrix} \sum_i X_i^2 & \sum_i X_i \\ \sum_i X_i & n \end{pmatrix}$$

where X_i is in our case $\mathbb{E}(X_i|Y_i, Z_i)$ and $\pi_X = \mathbb{P}(Y = 1|X)$. Taking the inverse at the null we have (using $\beta = 0, \pi_X = \bar{Y}$):

$$I^{-1}(\alpha, \beta) = \frac{1}{\bar{Y}(1-\bar{Y})(n\sum_i X_i^2 - (\sum_i X_i)^2)} \begin{pmatrix} n & -\sum_i X_i \\ -\sum_i X_i & \sum_i X_i^2 \end{pmatrix}$$

So the score variance is:

$$V = \bar{Y}(1-\bar{Y})(n\bar{X_i^2} - \bar{X_i}^2) = \frac{DH}{n} s_X^2$$

Fuzzy profile likelihood argument

We now show how the score variance is modified by the presence of fuzzy calls. The uncertainty on the calls adds a term to the score variance [2]. The problem is the dependence of f_j in β. If we note $g_j = L(Z|X, Y)$ we have, for cases:

$$\mathbb{E}(X|Y, Z) = \frac{\pi_j \phi_j g_j}{\sum_k \pi_k \phi_k g_k}$$

Assuming that ϕ and g remain constant (which is the case if the genotyping parameters $\gamma = (\theta, \phi)$ are not affected by variations of β), at the null $\beta = 0$ we have:

$$\begin{aligned} \frac{\delta \mathbb{E}(X|Y, Z)}{\delta \beta} &= \pi(1-\pi)\frac{\left[j^2 g_j \phi_j\right]\left[\sum_k \pi_k \phi_k g_k\right] - j g_j \phi_j \left[\sum_k k \phi_k g_k\right]}{\left[\sum_k \pi_k \phi_k g_k\right]^2} \\ &= \pi(1-\pi)\left[\sum_j j^2 f_j - \left[\sum_j j f_j\right]^2\right] \end{aligned}$$

The fuzzy calls add a term in the score variance. Interestingly, it is exactly the variance of X under the fuzzy posterior distribution. Of course if calls are known with certainty this variance is zero and one obtains the usual test statistic. If we denote this variance by s_i, the additional score variance is:

$$\pi(1-\pi)^2 s_i \text{ for cases and } \pi^2(1-\pi)s_i \text{ for controls}$$

The overall variance becomes:

$$V = \frac{DH}{n}\left[s_X^2 - \frac{(1-\bar{Y})\sum_{\text{cases}} s_i^2 + \bar{Y}\sum_{\text{controls}} s_i^2}{n}\right]$$

References

[1] Nelder JA, Mccullagh P (1983) Generalized Linear Models. Chapman and Hall.

[2] Louis TA (1982) Finding the observed information matrix when using the EM algorithm. Journal of the Royal Statistical Society Series B 44:226–233.
